# *Doryctopambolus* Nunes & Zaldívar-Riverón (Braconidae), a new neotropical doryctine wasp genus with propodeal spines

**DOI:** 10.3897/zookeys.223.3540

**Published:** 2012-09-26

**Authors:** Juliano Fiorelini Nunes, Alejandro Zaldívar-Riverón, Clóvis Sormus de Castro, Paul M. Marsh, Angélica Maria Penteado-Dias, Rosa Briceño, Juan José Martínez

**Affiliations:** 1 Fundação de Ensino Superior de Passos FESP/UEMG, Av. Juca Stockler, 1130, Bairro Belo Horizonte, CEP 37900-106, Passos, MG, Brasil; 2 Colección Nacional de Insectos, Instituto de Biología, Universidad Nacional Autónoma de México, 3er. Circuito Exterior, Cd. Universitaria, Ap. Postal 70-153, C. P. 04510, México D. F., México; 3 Programa de Pós-Graduação em Ecologia e Recursos Naturais. Universidade Federal de São Carlos Cx. Postal 676. CEP 13565-905, São Carlos, SP, Brasil; 4 P.O. Box 384, North Newton, Kansas 67117; USA; 5 Departamento de Ecologia e Biologia Evolutiva da Universidade Federal de São Carlos. Cx. Postal 676, CEP 13565-905, São Carlos, SP, Brasil; 6 Universidad Centroccidental “Lissandro Alvarado”, Decanato de Agronomía, Depto. de Ciencias Biológicas, Sección de Entomología, Tarabana, Cabudare, Estado de Lara, Venezuela; 7 CONICET-Museo Argentino de Ciencias Naturales “Bernardino Rivadavia”, Buenos Aires, Argentina

**Keywords:** Doryctinae, Pambolinae, new genus, Hymenoptera

## Abstract

The new Neotropical doryctine genus *Doryctopambolus* **gen. n.** is erected to contain *Doryctopambolus pilcomayensis* (van Achterberg & Braet, 2004**), comb. n.**, which was previously placed within *Pambolus* (Pambolinae), as well as three new species, *Doryctopambolus clebschi* **sp. n.**, *Doryctopambolus dominicanus* **sp. n.** and *Doryctopambolus sarochensis* **sp. n.** Members of this new genus are mainly characterised by the presence of at least one pair of conspicuous propodeal apico-lateral projections, which are similar to those present in all members of Pambolinae and in species of three Australasian doryctine genera. We generated DNA barcoding sequences for the three newly described species. We discuss the morphological similarity between species of the Australasian *Echinodoryctes* Belokobylskij, Iqbal & Austin and *Doryctopambolus*. A key for the described species of *Doryctopambolus* is provided.

## Introduction

The presence of propodeal or scutellar spines is a common feature found in species of a number of hymenopteran parasitoid families, including Eucharitidae, Figitidae, Ichneumonidae and Braconidae, among others. Within the cyclostome group of subfamilies belonging to Braconidae, the presence of acute tubercles or spines on the apical lateral region of propodeum has been considered as the main diagnostic character employed to distinguish members of the cosmopolitan Pambolinae (Quicke and van Achterberg 1990, van Achterberg 1995). However, species of three Australasian genera (*Fijispathius* Belokobylskij, Iqbal & Austin, *Echinodoryctes* Belokobylskij, Iqbal & Austin and *Ryukyuspathius* Belokobylsij; Belokobylskij et al. 2004, Belokobylskij 2008) and one described species of the Neotropical genus *Concurtisella* Roman (*Concurtisella bidens* Roman), all belonging to the cyclostome subfamily Doryctinae, also have these propodeal projections, indicating that this feature has actually evolved on separate occasions within Braconidae.

Among the 44 species of *Pambolus* described to date (Yu et al. 2005, Martínez et al. 2012), two species, *Pambolus pilcomayensis* van Achterberg & Braet and *Pambolus leponcei* van Achterberg & Braet, were described from northern Argentina by van Achterberg and Braet (2004). The authors placed these two species within Pambolinae since they have apical-lateral projections on the propodeum. However, a detailed examination of the description digital pictures of the holotype of *Pambolus pilcomayensis*, as well as additional specimens collected in Brazil and Argentina, have revealed that this species possesses the main diagnostic characters that distinguish members of Doryctinae, viz. fore tibial spines, double nodus and tip of ovipositor strongly sclerotized (Belokobylskij et al. 2004).

In this study we erect the new doryctine genus *Doryctopambolus* gen. n. to include *Pambolus pilcomayensis* (van Achterberg & Braet, 2004**),** comb. n. and three additional Neotropical species described herein. We also molecularly characterise the three newly described species with the DNA barcoding locus (Hebert et al. 2003) and discuss the apparently close relationship between species of this new genus and species of the Australian *Echinodoryctes* based on their shared external morphological features. 

## Methods

Specimens belonging to the new genus described in this study were collected in different localities in Argentina, Brazil, Dominican Republic and Venezuela. The Brazilian specimens assigned to *Pambolus pilcomayensis* were collected in a savannah area at Itumbiara-GO Brazil using Malaise traps. The Argentinian specimens from suburban areas of Buenos Aires, Argentina, were on the other hand collected with pit fall traps associated with riparian vegetation. Specimens assigned to the three newly described species were both collected with yellow pan traps in the Parque Nacional Cerro Saroche, Lara, Venezuela, and in Punta Cana and Parque Nacional Armando Bermudez, in Dominican Republic. All our examined material was compared with other Neotropical doryctine genera using Marsh’s (1997, 2002) keys.

Digital colour photographs of all the species included in this work were taken with a Leica® Z16 APO-A stereoscopic microscope and a Leica® DFC295/DFC290 HD camera, and were edited using the Leica Application Suite® program. Digital scanning electron microscope (SEM) photographs of *Pambolus pilcomayensis* were taken with a FEI QuantaTM 250 SEM in low vacuum mode. The specimens examined in this study are deposited at Universidade Federal de São Carlos – Departamento de Ecologia e Biologia Evolutiva, Brazil (DCBU), Colección Nacional de Insectos, Instituto de Biología, Universidad Nacional Autónoma de México (CNIN-IB UNAM), Museo Argentino de Ciencias Naturales “Bernardino Rivadavia”, Buenos Aires, Argentina (MACN), and Museo de La Plata, Argentina (MLP). Terminology for body structure, sculpture and wing venation features follows Marsh (2002).

DNA sequences belonging to the barcoding locus [~650 bp of the cytochrome oxidase I (COI) mitochondrial DNA gene; Hebert et al. 2003] were generated for specimens belonging to the new genus using the DNA extraction and amplification protocols employed in a previous study (Ceccarelli et al. 2012). Genetic distances were calculated for the sequenced specimens using the K2P distance model with PAUP* version 4.10b (Swofford 2002).

## Taxonomy

### Doryctopambolus

Nunes & Zaldívar-Riverón gen. n.

urn:lsid:zoobank.org:act:2C42D9FA-392C-4F6E-8A6E-CA00EAE711C0

http://species-id.net/wiki/Doryctopambolus

**Figure 1.**
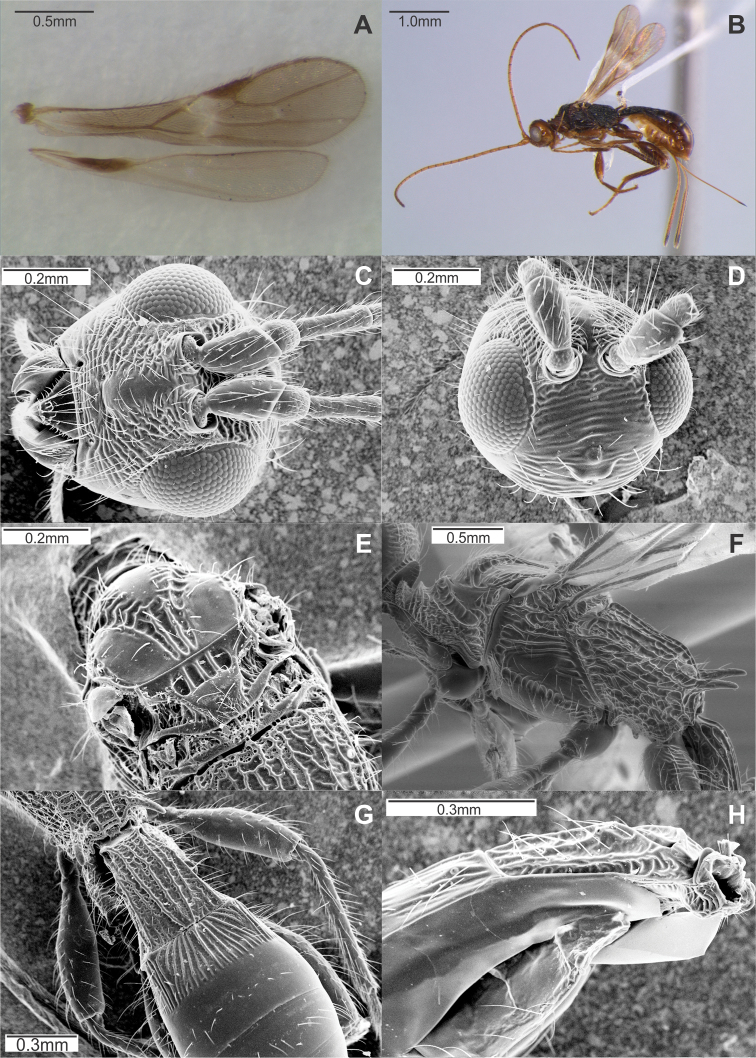
*Doryctopambolus pilcomayensis* comb. n.: **A** male wings **B** female habitus, lateral view **C** head, frontal view **D** head, dorsofrontal view **E** mesonotum, dorsal view **F** mesosoma, lateral view **G** metasoma, dorsal view **H** fore segment with acrosternite, latero-ventral view.

Figures 1A–H 3A–H 4A–E

#### Type species.

*Pambolus pilcomayensis* van Achterberg & Braet, 2004

#### Diagnosis.

**Figure 2.**
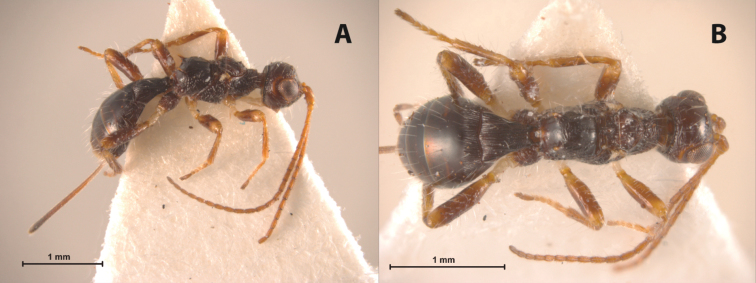
*Echinodoryctes tetraspinosus* (*paratype*): **A** female habitus, lateral view **B** female habitus, dorsal view.

Species of*Doryctopambolus* can be distinguished from members of most doryctine genera except *Concurtisella bidens*, *Echinodoryctes*, *Fijispathius* and *Ryukuspathius* by having the propodeum with at least one pair of conspicuous apico-lateral projections. Species of *Doryctopambolus* and *Concurtisella bidens* are the only Neotropical doryctine taxa reported to have these projections, though they mainly differ by their first subdiscal cell (open at apex in *Doryctopambolus*, closed in *Concurtisella bidens*) and ovipositor length (about the same length as metasoma in *Doryctopambolus*, longer than body in *Concurtisella bidens*). *Doryctopambolus* differs from the Australasian *Fijispathius* and *Ryukyuspathius* mainly by the fore wing first subdiscal cell open at apex (closed in the later two genera) and the first metasomal segment not petiolate (basal sternal plate at most 0.5 lenght of first tergite in *Doryctopambolus*, 0.65 to 0.7 in *Fijispathius* and *Ryukyuspathius*). *Doryctopambolus* is morphologically similar to the Australian *Echinodoryctes* (figs 2A–B). However, species of *Doryctopambolus* differ from those of *Echinodoryctes* by having partially reduced to well-developed wings (micropterous in *Echinodoryctes*), propodeum evenly curved and strongly rugose-areolate (globose and mostly smooth in *Echinodoryctes*), hind coxa without basoventral tubercle and all femora without dorsal protuberances (both present in *Echinodoryctes*).

#### Description.

Small size, 2.2–3.6 mm; black to light brown species. *Head*: head globose; antennal sockets distinctly separated from each other by at least 0.5 times its diameter; frons almost flat, without median carina or furrows; ocelli arranged in equilateral triangle; eye with distinct and sparse setae; gena and temple smooth; malar suture absent; first flagellomere slightly shorter than scape and pedicel combined, slightly longer than second flagellomere; antenna with 16–28 antennomeres; occipital carina meeting hypostomal carina before mandible. *Mesosoma*: Length of mesosoma about two times its maximum width; neck of pronotum fairly long; pronotal crest conspicuous; mesoscutum declivous anteriorly; mesoscutal lobes smooth and polished medially; notauli complete and strongly impressed; scutellar sulcus deep, with its height 0.8–0.9 times height of scutellar disc; precoxal sulcus complete and scrobiculate, as long as mesopleuron; prepectal carina coarse and complete; propodeum evenly curved and strongly rugose-areolate, with at least one pair of conspicuous apico-lateral projections; propodeal bridge absent. *Legs*: fore tibia with a row of 7–8 stout spines; middle tibia without spines; femora without dorsal protuberances; hind coxa without basal tubercle. *Wings*: partially reduced to well-developed wings; fore wing veins r-m and 2RS present; m-cu arising interstitial or slightly antefurcal with vein 2RS, cu-a distinctly postfurcal with vein 1M; first subdiscal cell open at apex; hind wing vein M+CU equal length of vein 1M; cu-a present, m-cu absent; stigma present on male hind wing. *Metasoma*: length of first metasomal tergum 1.3–1.6 times its apical width, apical width about 2.0–2.3 times basal width; basal sternal plate (acrosternite) about 0.33–0.5 times length of tergum; suture between second and third metasomal tergites absent; second metasomal tergite at least sculptured basally; third metasomal tergite usually smooth, sometimes sculptured basally; remaining metasomal tergites entirely smooth and polished; ovipositor about same length of metasoma.

#### Distribution.

Neotropical. Known from central Argentina to northern Venezuela, and from Dominican Republic in the Caribbean.

#### Comments.

Three new species of *Doryctopambolus* and *Doryctopambolus pilcomayensis* comb. nov. are described and redescribed in this study, respectively. Four additional species belonging to this genus, two from Cerro Saroche, Lara, Venezuela, and two from Argentina, were also identified. The two Argentinian species could not be described due to their bad state of preservation.Of the two species from Venezuela, one was represented by a single male and the other one by an incomplete female, and their allospecificity was corroborated with DNA barcoding sequences (DNA voucher nos. DORYC239, 274; GenBank accession numbers JN266989, JN267020). The Parque Nacional Cerro Saroche is a natural reserve of about 32,294 h mainly composed of xeric vegetation with deciduous and semideciduous shrubs (Inparques 1992). The doryctine fauna from this reserve has been previously reported by Briceño et al. (2009), and includes some rarely collected genera such as *Verae* Marsh, *Coiba* Marsh and *Hecabolus* Curtis.

Our morphological observations revealed that the species of *Doryctopambolus* share various external morphological features with the two described species of the endemic Australian *Echinodoryctes* (figs 2A–B), including a similar body habitus, at least one pair of apico-lateral propodeal projections and the second metasomal tergite at least partially sculptured. Further morphological and molecular studies will confirm whether or not species of these two genera are congeneric.

#### Etymology.

Combination from the doryctine generic names *Doryctes* Haliday, 1836 and *Pambolus* Haliday, 1836, since the type species of this new genus was previously placed within *Pambolus*. Gender is masculine.

### Doryctopambolus pilcomayensis

(van Achterberg & Braet, 2004) comb. n.

Figures 1A–H

Pambolus pilcomayensis van Achterberg & Braet 2004: pp. 341–344.

#### Diagnosis.

This species distinguishes from *Doryctopambolus sarochensis* sp. n. by having one pair of propodeal apico-lateral projections (two pairs in *Doryctopambolus sarochensis* sp. n.) and third metasomal tergite entirely smooth (costate baso-laterally). *Doryctopambolus pilcomayensis* differs from *Doryctopambolus dominicanus*sp. n. and *Doryctopambolus clebschi* sp. n. by having the vertex striate (smooth in *Doryctopambolus dominicanus* sp. n. and *Doryctopambolus clebschi* sp. n.), first metasomal tergite not petiolate (at least 0.5 of tergum) and second metasomal tergite entirely sculptured (mostly smooth).

#### Redescription.

Females. Body length 3.0–3.6 mm, fore wing 2.0 mm, ovipositor 1.8 mm. *Colour*: head light to dark brown, antenna light brown, malar space light brown to yellowish, mandible and palpi light brown; propleuron and pronotum light brown, remainder of mesonotum brown to black; wings slightly infuscate, narrow banded with brown veins; metasoma brown to black, first two metasomal tergites darker; legs brown to dark brown, fore and middle coxae, trochanter, trochantellus and tarsi light brown to pale yellow; ovipositor and sheaths light brown with dark apex. *Head*: antero-dorsal surface of eyes bordered by groove; eyes setose; face costate-rugose laterally, central area slightly swollen and smooth; clypeus costate-rugose; malar space 0.8–1.0 times eye height; frons and vertex striate; temple in dorsal view 0.7 times eye width; antenna with 26 antennomeres. *Mesosoma*: length of mesosoma 2.0 times its maximum height; pronotum rugose; pronotal groove wide and scrobiculate laterally, dorsally smooth; propleuron costate-rugose; notauli deep and scrobiculate, meeting scutellum with parallel carinae; scutellum smooth and setose; scutellar sulcus with three parallel carinae; mesopleuron porcate dorsally, smooth near precoxal sulcus and ventrally; ventral portion of mesopleuron separated by shallow crenulate median groove with some rugosities near middle coxae; propodeum with one pair of conspicuous apico-lateral projections, longer than first flagellomere; median carina present only basally. *Legs*: hind coxa costate to costate-rugose dorsally. *Wings*: fore wing length 3.7–4.8 times its maximum width, r:3RSa:3RSb = 2:8-12:18-32; 2RS:3RSa:r-m = 6:8-12:2-3; m-cu arising interstitial or slightly antefurcal to vein 2RS; 1cu-a interstitial with 1M. *Metasoma*: length of first metasomal tergite 1.3 times its apical width, apical width about 2.0 times basal width costate-rugose, with two or three longitudinal carinae reaching apex of tergum; second metasomal tergite strongly costate; remaining metasomal tergites smooth and polished; basal sternal plate (acrosternite) about 0.33 times length of tergum; ovipositor nearly as long as metasoma.

**Males.** Essentially as females; body length 2.1–3.0 mm; body brown, with antennae, trochanter, trochantellus, tarsi, fore and middle coxae light brown to yellowish; face, frons and vertex slightly sculptured; stigma present in hind wing.

#### Material examined.

Twenty-two specimens (DCBU, MACN, MLP). **Brazil:** Goiás State (GO), Itumbiara, Malaise trap, C.H. Marchiori col.: one female 24-I-1998, three males 28-I-1998, one female and one male 31-I-1998, two males 7-II-1998, three females and one male 28-II-1998, one female and one male 7-III-1998, one female 1-IV-1998, one female and one male 15-IV-1998, one female 08-XI-1998 (DCBU); Mato Grosso do Sul State (MS), Bodoquena, Parque Nacional da Serra da Bodoquena, Faz. Pitangueiras, Bandeja Am. 21 (yellow pan trap), Crepaldi RA et al. col: one female V-2009 (DCBU). **Argentina:** Misiones Province, Estación Experimental Loreto, Dr. A. Ogloblin: one female 15-VI-1930 (MLP); Buenos Aires Province, Reserva Ecológica de Vicente López, 34.492°S, 58.480°W: one male 16-I-2011 (MACN); one male, no data (MLP).

#### Distribution.

Southern Brazil to Argentina.

#### Comments.

The presence of *Doryctopambolus pilcomayensis* near Buenos Aires can be explained by the courses of the Parana and Uruguay basins, which carry downstream many plant and animal species from tropical areas to higher latitudes.

### Doryctopambolus clebschi

Zaldívar-Riverón & Martínez sp. n.

urn:lsid:zoobank.org:act:0992A72F-1163-48FB-A1BF-D5E59F7D117F

http://species-id.net/wiki/Doryctopambolus_clebschi

Figures 3A–C

#### Diagnosis. 

*Doryctopambolus clebschi* is morphologically similar to *Doryctopambolus dominicanus* sp. n. However, *Doryctopambolus clebschi* differs from the latter species by having the face smooth medially and slightly rugose laterally (entirely rugose in *Doryctopambolus dominicanus* sp. n.), mesopleuron mostly smooth (mostly smooth-rugose), first metasomal tergite mostly smooth apically (mostly sculptured apically), and second metasomal tergite mostly smooth, slightly costate baso-laterally (costate basally). These two species distinguish from the remaining two described species of *Doryctopambolus*, *Doryctopambolus pilcomayensis* comb. n. and *Doryctopambolus sarochensis* sp. n., by having the vertex smooth (always sculptured in the latter two species), first metasomal tergite distinctly petiolate (not petiolate), and second metasomal tergite partially smooth (always entirely sculptured).

#### Description.

Female**.** Body length 2.8 mm; fore wing 2.3 mm; ovipositor 1.7 mm. *Colour*: head brown, antennae brown, turning honey yellow to apex, palpi yellow; mesosoma and first metasomal tergites brown, second metasomal tergite honey yellow, remaining metasomal tergites light brown; wings hyaline; veins and stigma light brown; legs light brown to honey yellow; ovipositor and sheaths light brown to brown, apex strongly sclerotised and dark. *Head*: antenna with 16-17 antennomeres; eyes small, ovoid and setose; face smooth and glabrous medially, slightly rugose and pilose laterally, median area not swollen; clypeus slightly rugose; malar space 0.4 times eye height; frons and vertex smooth; temple in dorsal view 2.7 times eye width. *Mesosoma*: 1.8 times longer its maximum height; pronotum rugose laterally; pronotal groove wide and scrobiculate; propleuron slightly rugose; mesoscutal lobes entirely smooth and polished, sparsely pilose; notauli wide, deep and scrobiculate, meeting before scutellum in a longitudinally costate area; scutellum smooth, sparsely setose; scutellar sulcus with three parallel carinae; height of scutellar sulcus 0.8 times height of scutellar disc; subalar sulcus wide and scrobiculate, joining mesopleural sulcus, remaining area of mesopleuron mostly smooth and polished, slightly rugose near mesopleural sulcus; venter of mesopleuron smooth; propodeum with one pair of long and sharp apico-lateral projections, shorter than first flagellomere. *Legs*: hind coxa mostly smooth, slightly rugose ventrally. *Wings*: fore wing length 3.6 times its maximum width, r:3RS:3RSb = 2:3:10; 2RS:3RSa:r-m = 4:3:2; m-cu arising antefurcal with vein 2RS; 1cu-a interstitial with 1M; hind wing vein M + CU about equal length of vein 1M. *Metasoma*: length of first metasomal tergum 1.5 times its apical width, apical width about 2.3 times basal width, first metasomal tergite costate basally and medially, smooth apically; second metasomal tergite mostly smooth and polished, slightly costate baso-laterally; remaining metasomal tergites smooth and polished; basal sternal plate (acrosternite) about 0.5 times length of tergum; ovipositor about 1.1 times longer than metasoma.

**Variation.** Females. Body length 2.6–2.8 mm.

**Males.** Unknown.

**Figure 3.**
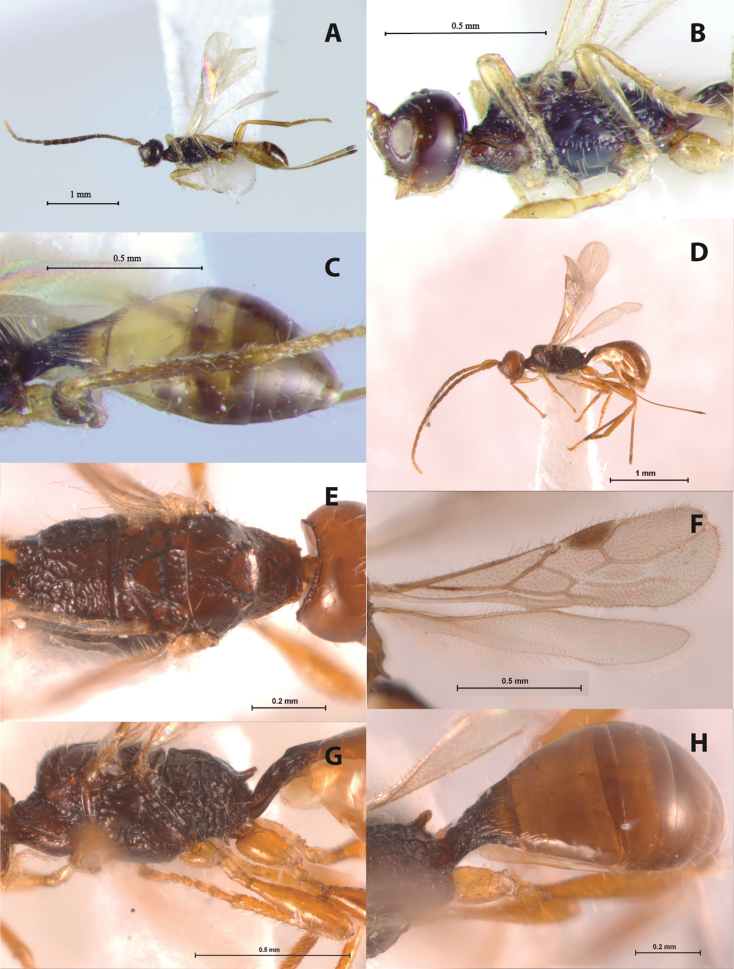
**A–C** *Doryctopambolus clebschi* sp. n. (*holotype*): **A** female habitus, lateral view **B** head and mesosoma, lareal view **C** metasoma, latero-dorsal view. **D–H** *Doryctopambolus dominicanus*sp. n. (*holotype*) **D** female habitus, lateral view; **E** mesosoma, dorsal view **F** female wings **G** mesosoma and first metasomal segment, lateral view **H** metasoma, dorsal view.

#### Material examined.

*Holotype*. Female (CNIN IB-UNAM). **Dominican Republic:** A. Bermudez NP, La Ciénega, Telostablones, 19.066°N, 70.863°W, 15-16-IX-2009, sweep, H. Clebsch col. (DNA voucher no. CNIN370; GenBank accession number JN870294). *Paratype*. One female (CNIN IB-UNAM), same data as holotype.

#### Distribution.

Dominican Republic.

#### Etymology.

This species is named in honour to our good friend and colleague Hans Clebsch, who collected the specimens assigned to this species.

### Doryctopambolus dominicanus

Zaldívar-Riverón & Martínez sp. n.

urn:lsid:zoobank.org:act:99C34338-DB4F-4AE2-A662-4DDA9957BA43

http://species-id.net/wiki/Doryctopambolus_dominicanus

Figures 3D–H

#### Diagnosis.

See *D. Clebschi*.

#### Description.

Female**.** Body length 2.7 mm; fore wing 1.8 mm; ovipositor 1.3 mm. *Colour*: head light brown, antennae light brown, turning honey yellow to apex, palpi yellow; mesosoma and first and basal two thirds of second metasomal tergites dark brown; wings hyaline; veins and stigma light brown; legs light brown to honey yellow; ovipositor and sheaths honey yellow, apex strongly sclerotised and dark. *Head*: 16 flagellomeres (one antenna broken, with only 11 flagellomeres; eyes ovoid, small and setose; face rugose, median area not swollen; clypeus slightly rugose; malar space 0.4 times eye height; frons and vertex smooth; temple in dorsal view 2.0 times eye width. *Mesosoma*: 1.7 times longer its maximum height; pronotum rugose-coriaceous laterally; pronotal groove wide and scrobiculate; propleuron slightly rugose; mesoscutal lobes entirely smooth and polished; notauli wide, deep and scrobiculate, meeting before scutellum in a longitudinally costate area; scutellum smooth, with some setae; scutellar sulcus with five carinae; height of scutellar sulcus 0.9 times height of scutellar disc; subalar sulcus wide, deep and scrobiculate, joining mesopleural sulcus, remaining area of mesopleuron smooth, rugose near precoxal sulcus; venter of mesopleuron smooth; propodeum with one pair of long and sharp apico-lateral projections, slightly shorter than first flagellomere. *Legs*: hind coxa mostly smooth, slightly rugose ventrally. *Wings*: fore wing length 3.8 times its maximum width, r:3RSa:3RSb = 1:2:4; 2RS:3RSa:r-m = 2:2:1; m-cu arising antefurcal with vein 2RS; 1cu-a slightly postfurcal with 1M; hind wing vein M + CU about equal length of vein 1M. *Metasoma*: length of first metasomal tergum 1.6 times its apical width, apical width about 2.1 times basal width, first metasomal tergite costate with slightly rugose microsculpture; basal third of second metasomal tergite costate, apical two thirds smooth and polished; remaining metasomal tergites smooth and polished; basal sternal plate (acrosternite) about 0.5 times length of tergum; ovipositor 0.9 times as long as metasoma.

#### Material examined.

*Holotype*.Female (CNIN IB-UNAM). **Dominican Republic:** Punta Cana (Rural), Malaise trap, Masner col. (DNA voucher no. CNIN749; GenBank accession number JX507736).

#### Distribution.

Dominican Republic.

#### Etymology.

The name of this species refers to the country where the holotype was collected, Dominican Republic.

### Doryctopambolus sarochensis

Nunes & Zaldívar-Riverón sp. n.

urn:lsid:zoobank.org:act:9C8C7812-2519-4849-A2CB-D3EED92F7FFB

http://species-id.net/wiki/Doryctopambolus_sarochensis

Figures 4A–E

#### Diagnosis.

*Doryctopambolus sarochensis* sp. n. distinguishes from the remaining species of the genus by having the vertex striate-rugose (striate in *Doryctopambolus pilcomayensis* comb. n. and smooth in the remaining two species), two pairs of propodeal apico-lateral projections, the basal ones small and blunt and the second pair long and distinctly truncate, and third metasomal tergite costate baso-laterally (entirely smooth in the other species).

#### Description.

Female**.** Body length 3.0 mm; fore wing 2.0 mm; ovipositor 1.7 mm. *Colour*: head brown, malar space honey yellow, antenna honey yellow, turning brown at apex, mandible honey yellow, palpi yellow; mesosoma and metasoma dark brown to black; wings slightly infuscate; veins and stigma brown; fore leg honey yellow; middle leg honey yellow, with femur light brown; hind coxa and femur brown, trochanter, trochantellus, tibia and tarsi honey yellow; ovipositor and sheaths honey yellow, apex strongly sclerotised and dark. *Head*: 23 antennomeres; eyes setose; face striate medially, striate-rugose laterally, with median area slightly swollen; clypeus costate-rugose; malar space 0.6 times eye height; frons and vertex striate-rugose; temple in dorsal view 0.8 times eye width. *Mesosoma*: two times longer its maximum height; pronotum rugose laterally; pronotal groove wide and scrobiculate; propleuron costate-rugose; mesoscutal lobes mostly smooth and shinning, rugose along notauli and at lateral edges; notauli narrow and scrobiculate, meeting before scutellum in a longitudinally costate-rugose area; scutellum smooth, with some setae; scutellar sulcus with three parallel carinae; height of scutellar sulcus 0.8 times height of scutellar disc; subalar sulcus wide, deep and scrobiculate, joining mesopleural sulcus in an inverted “V” shape; mesopleuron porcate dorsally, porcate-rugose laterally, smooth ventrally; venter of mesopleuron smooth; propodeum with two pairs of apico-lateral projections, the most basal one small and blunt, the apical one long and distinctly truncate, longer than first flagellomere. *Legs*: hind coxa strongly costate-rugose dorsally, poorly sculptured ventrally. *Wings*: fore wing length 3.8 times its maximum width, r:3RS:3RSb = 3:6:22; 2RS:3RSa:r-m = 8:6:5; m-cu arising antefurcal with vein 2RS; 1cu-a interstitial with 1M. *Metasoma*: length of first metasomal tergite 1.5 times its apical width, apical width about 2.0 times basal width, costate rugose with two dorsal carinae converging at apex; second metasomal tergite strongly costate; third metasomal tergite mostly smooth, costate baso-laterally, remaining metasomal tergites smooth and polished; basal sternal plate (acrosternite) about 0.33 times length of tergum; ovipositor as long as metasoma.

**Variation.** *Females*. Body length 2.7–3.0 mm; antenna with 22–23 antennomeres. *Males*. Unknown.

**Figure 4.**
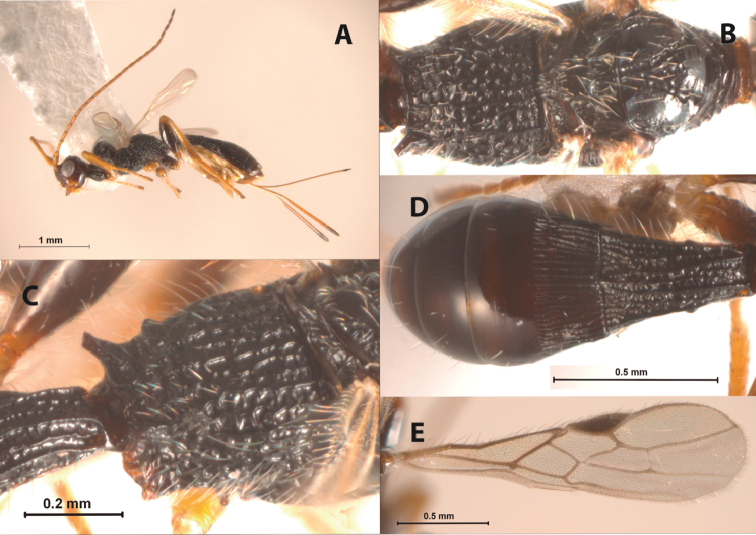
*Doryctopambolus sarochensis* sp. n.(*holotype*): **A** female habitus, lateral view **B** mesosoma, dorsal view **C** propodeum, dorsolateral view **D** metasoma, dorsal view **E** fore wing.

#### Material examined.

*Holotype*.Female (CNIN IB-UNAM). **Venezuela:** Lara, Parque Nacional Cerro Saroche, Cañaote #3, 10°11'083"N, -69°26'013"W; 1000 msnm; R. Briceño, R. Paz, W. Román, D. Torres colls.; (DNA voucher no. DORYC-236; GenBank accession number JN266986). *Paratype*. Onefemale (CNIN IB-UNAM), same data as holotype (DNA voucher no. DORYC-238; GenBank accession number JN266988).

#### Distribution.

NortheastVenezuela.

#### Etymology.

The name of this species refers to the place where the type material was collected.

#### Additional examined material

**.** *Echinodoryctes tetraspinosus* Belokobylskij, lqbal & Austin. One specimen. *Paratype*. Female. Australia: Prevelly Park, W of Margaret River, 1 nov. 1984 W.A., I. & N. Lawrence, in marri nuts on ground. *Doryctopambolus* cf. *pilcomayensis*: *Doryctopambolus* sp. 1: one female (MLP): **Argentina**, Misiones, Estación Experimental Loreto, Dr. A. Ogloblin, 25-VI-1931; one male (MLP): Argentina, Misiones, Estación Experimental Loreto, Dr. A. Ogloblin, 8-VI-1933; *Doryctopambolus* sp. 2: one male (MACN): **Argentina**, Ciudad Autónoma de Buenos Aires, Reserva Ecológica Costanera Sur, trampa de caída, Mamani, Turienzo, Zapata, 19-I-2009; *Doryctopambolus* sp. 3 (CNIN IB-UNAM): **Venezuela**, Lara, Parque Nacional Cerro Saroche, Cañaote, 10°11'083"N, 69°26'013"W; 1000 m; R. Briceño, R. Paz, W. Román, D. Torres colls. (DNA voucher no. DORYC-239; GenBank accession number JN266989); *Doryctopambolus* sp. 4 (CNIN IB-UNAM): **Venezuela**, Carabobo, Palmichal, 10.2859N, 68.2399W, 931 m, 30-31-iii-07, YPT/64 plates, shade coffee/Orange grove plantations, H. Clebsch col. (DNA voucher no. DORYC-274; GenBank accession number JN267020).

#### Genetic distances.

Interspecific variation of the barcoding locus among the examined species of *Doryctompambolus*, *Doryctopambolus clebschi* (two specimens), *Doryctopambolus dominicanus* (one specimen), *Doryctopambolus sarochensis* (two specimens) and the two undescribed species from Venezuela (one specimen each), was congruent with the interspecific variation observed in other braconid genera, ranging from 4.2 to 14%. The lowest interspecific genetic distance occurred between specimens of the two Dominican species, *Doryctopambolus clebschi* and *Doryctopambolus dominicanus*.

##### Key to described species of *Doryctopambolus*

**Table:** 

1	Two pairs of propodeal apico-lateral projections	*Doryctopambolus sarochensis* Nunes & Zaldívar-Riverón
–	One pair of propodeal apico-lateral projections	2
2	Vertex striate; first metasomal tergite weakly petiolate (about 0.3 of tergum); second metasomal tergite entirely sculptured	*Doryctopambolus pilcomayensis* (van Achterberg & Braet)
–	Vertex smooth; first metasomal tergite distinctly petiolate (at least 0.5 of tergum); second metasomal tergite mostly smooth	3
3	Face entirely rugose, mesopleuron mostly smooth; 3RS of fore wing about 2.0 times 3RSb; first metasomal tergite entirely sculptured; second metasomal tergite costate on basal third, remaining area smooth	*Doryctopambolus dominicanus* Zaldívar-Riverón & Martínez
–	Face smooth medially, slightly rugose laterally; mesopleuron mostly smooth-rugose; 3RS of fore wing about 3.0 times 3RSb; first metasomal tergite smooth apically; second metasomal tergite mostly smooth, slightly costate basolaterally	*Doryctopambolus clebschi* Zaldívar-Riverón & Martínez

## Supplementary Material

XML Treatment for Doryctopambolus

XML Treatment for Doryctopambolus pilcomayensis

XML Treatment for Doryctopambolus clebschi

XML Treatment for Doryctopambolus dominicanus

XML Treatment for Doryctopambolus sarochensis
